# Breast cancer arising within fibroadenoma: collective analysis of case reports in the literature and hints on treatment policy

**DOI:** 10.1186/1477-7819-12-335

**Published:** 2014-11-10

**Authors:** Yu-Ting Wu, Shou-Tung Chen, Chih-Jung Chen, Yao-Lung Kuo, Ling-Ming Tseng, Dar-Ren Chen, Shou-Jen Kuo, Hung-Wen Lai

**Affiliations:** Endoscopic and Oncoplastic Breast Surgery Center, Comprehensive Breast Cancer Center, Changhua Christian Hospital, 135 Nanhsiao Street, Changhua City, Changhua County 500 Taiwan; Division of General Surgery, Changhua Christian Hospital, 135 Nanhsiao Street, Changhua City, Changhua County 500 Taiwan; Department of Surgery, Comprehensive Breast Cancer Center, Changhua Christian Hospital, 135 Nanhsiao Street, Changhua City, Changhua County 500 Taiwan; Department of Surgery, Sinying Hospital, Ministry of Health and Welfare, 73 Xinyi Street, Xinying Dist, Tainan City, 730 Taiwan; Department of Surgical Pathology, Changhua Christian Hospital, 135 Nanhsiao Street, Changhua City, Changhua County 500 Taiwan; School of Medicine, Chung Shan Medical University, Taichung, No. 110, Sec. 1, Jianguo N. Rd, Taichung City, 40201 Taiwan; Department of Medical Technology, Jen-Teh Junior College of Medicine, Nursing and Management, No 79-9, Sha-Luen Hu, Xi-Zhou Li, Hou-Loung Town, Miaoli County, Taiwan; Division of General Surgery, Department of Surgery, National Cheng-Kung University Hospital, Tainan and Dou-Liou branches, No.138, Sheng Li Road, Tainan 704, Taiwan and No.345, Zhuangjing Rd, Douliu City, Yunlin County 64043 Taiwan; Division of Plastic and Reconstructive Surgery, Department of Surgery, National Cheng-Kung University Hospital, Tainan and Dou-Liou branches, No.138, Sheng Li Road, Tainan 704, Taiwan and No.345, Zhuangjing Rd, Douliu City, Yunlin County 64043 Taiwan; Division of General Surgery, Department of Surgery, Taipei Veterans General Hospital, No.201, Sec. 2, Shipai Rd., Beitou District, Taipei City, 11217 Taiwan; School of Medicine, National Yang Ming University, Taipei, No.155, Sec.2, Linong Street, Taipei, 112 Taiwan

**Keywords:** Breast cancer, Fibroadenoma, Hormone therapy, Radiotherapy

## Abstract

**Background:**

Breast cancer arising within a fibroadenoma (BcaFad) is rare; the rate varies from 0.002% to 0.125% in fibroadenoma specimens. Owing to its rarity, the clinicopathologic feature and treatment principle of BcaFad is still not clear. Therefore, the aim of this study was to perform a collective analysis of case reports in the literature to identify the characteristics and optimal treatment for BcaFad.

**Methods:**

We analyzed an aggregated sample of 30 patients with BcaFad from case reports in the literature (*n* =24 cases) and our present study (*n* =6 cases). We collected and analyzed the clinicopathologic features and prognoses of patients with BcaFad, as well as treatments they received.

**Results:**

The patients’ mean age at diagnosis was 46.9 years. Twenty BcaFad patients (66.7%) received breast-conserving surgery (BCS), and nine other patients (30.0%) were treated with mastectomy. The rate of lymph node metastasis in BcaFad patients was 23.8%. The breakdown of the histological types of BcaFad was invasive ductal carcinoma (53.3%), followed by ductal carcinoma *in situ* (23.3%), lobular carcinoma *in situ* (16.7%) and invasive lobular carcinoma (13.3%). More than half of patients with positive hormone receptor status received hormone therapy. Most BcaFad patients with lymph node metastases received chemotherapy, and 20.0% of BcaFad patients treated with BCS received further radiotherapy. Only one patient had recurrence after surgery, and another had lung metastasis when diagnosed with BcaFad.

**Conclusions:**

Most BcaFad patients could be managed by BCS. Adjuvant radiotherapy could be performed, but was not mandatory. Chemotherapy should be considered as a treatment option in the presence of lymph node metastasis.

## Background

Fibroadenoma is the most common cause of discrete breast lumps in young females [1, 2] and occurs in 25% of asymptomatic women. Benign, asymptomatic fibroadenomas usually can be managed with nonoperative follow-up [3–5]. Symptomatic, progressively enlarging masses or atypical presentations, however, may warrant surgical excision.

Although very rare, breast cancer arising within fibroadenoma (BcaFad) can still be found. The incidence of BcaFad ranges from 0.125% to 0.02%, according to different reports [6–8]. In such circumstances, carcinomas *in situ* (66.9% lobular carcinoma *in situ* (LCIS) and 12.4% ductal carcinoma *in situ* (DCIS)) are found to be more common than invasive carcinomas (11% invasive ductal carcinoma (IDC) and 3.4% invasive lobular carcinoma (ILC)) [9]. The optimal management of patients with BcaFad is still not clear. Little information is available in current breast cancer management consensus guidelines [10–13]. It is unknown whether such patients should be treated similarly to those with breast cancer.

Owing to the rarity of BcaFad cases, clinicians in individual medical institutions have very little experience in treating these patients. Therefore, a collective case study is needed for more information about the optimal management of BcaFad. In this article, we present our experience with six BcaFad cases and used a systematic literature review process to find some characteristics of and hints for the optimal care of these patients.

## Methods

To further elucidate the characteristics and optimal management of patients with BcaFad, we performed a collective analysis of case reports in the literature and our six cases. The literature review was performed through PubMed searches to collect English-language articles about BcaFad published between January 1986 and January 2013. The keywords used in the search use were "fibroadenoma", "carcinoma", "breast", "ductal" and "lobular".

The literature search was limited to studies focused on BcaFad with descriptions of diagnosis and/or treatment. The investigated parameters included age, gender, tumor size, diagnostic image, operation method, histology, hormone receptor status (including estrogen receptor (ER) and progesterone receptor (PR)), Human epidermal growth factor receptor 2 (HER2) status, chemotherapy and radiation therapy. The searches were limited to human studies. Articles were excluded if they did not present BcaFad, patient information or treatment details. Articles published in any language other than English were also excluded.

Demographic data collected and analyzed included age, gender, size of the primary lesion, duration of follow-up, image findings, fine-needle aspiration cytology or core-needle biopsy results, pathology, operation method, postoperative therapy and recurrence or metastasis of the tumor.

## Results

**Figure 1:**
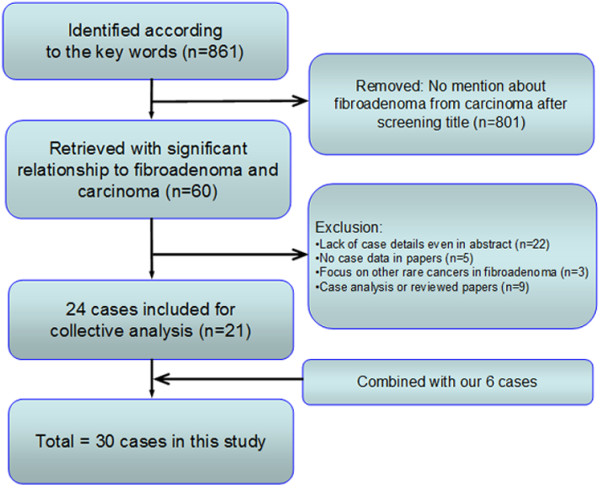
**Flowchart of the search strategy.** We initially identified 861 articles in the databases searched according to the keywords used. We excluded 801 articles after title screening because they did not mention fibroadenoma-related carcinoma, leaving 60 articles retrieved that described a significant relationship between fibroadenoma and carcinoma. We excluded 22 articles for lack of case details, even in the abstracts; 5 articles for no case data in the main text; 3 articles for focusing on other rare cancers linked to fibroadenoma; and 9 articles because they were case analyses or reviews. Thus, 24 cases from 21 studies were included, together with our 6 cases, which gave us a total of 30 cases for the final analysis.

In our literature review, we collected 24 cases with detailed information from 21 English-language articles. After combining these with our 6 cases, the study population comprised 30 BcaFad patients (Figure 1 and Table 1). All of the patients were female, and their mean age at diagnosis was 46.9 years old (range, 27 to 80 years old). The mean duration from the finding of fibroadenoma to surgical excision was 61.2 months (range, 0.5 to 600 months). The mean tumor size of the fibroadenoma was 2.46 cm (range, 0.8 to 5.1 cm). Most of the cancers found inside the breast fibroadenoma were *in situ* carcinoma or early breast cancer with minimal or small tumor size. The distribution of tumor locations was the right side (53.3%), followed by the left side (40.0%) and bilateral breast (3.3%). The clinicopathologic data of these 30 patients are summarized in Table 1.

**Table 1:** **Demographic data from identified studies in the literature and our cases** ^**a**^

Patient	Study	Age (yr)	Tumor size (cm)	Noted tumor duration (months)	Histology	TNM stage	Surgery	LN dissection	LN metastasis	Receptor status	C/T	H/T	R/T	Outcome
1	Kurosumi *et al*., 1994 [14]	42	2 × 3	21	IDC		Ex	A						
2	Sarela *et al*., 1995 [15]	56	2 × 3	2	LCIS + IDC		MT	A	P					
3	Shah *et al*., 1999 [16]	45	2.5 × 2	3	LCIS	TisN0M0	Ex		N					
4	Yano *et al*., 2001 [17]	54	2		ILC	TisN0M0	Ex	A	N					
5	Kuijper *et al*., 2002 [18]	46	Multiple		DCIS + LCIS	TisN0M0	MT							
6	Abe *et al*., 2004 [19]	42	5.1 × 4.4	60	IDC		MT	A	P	ER-PR+	Yes	Yes	No	
7	Stafyla *et al*., 2004 [20]	27	3.4		LCIS	TisN0M0	Ex		N					
8	Shin *et al*., 2006 [21]	51	1.5 × 1	12	IDC		MT	S	N	ER+PR+				
9	Yuen *et al*., 2007 [22]	39	1.1		ILC		BCS							
10	Yuen *et al*., 2007 [22]	45	0.8		ILC		BCS							
11	Borecky and Rickard, 2008 [23]	64	1.2		IDC	T1bN0M0	BCS	S	N	ER+PR+				
12	Borecky and Rickard, 2008 [23]	80	4.5	600	IDC		BCS	S	N					
13	Borecky and Rickard, 2008 [23]	53	1.7	12	DCIS	TisN0M0	Ex	S	N					
14	Gashi-Luci *et al*., 2009 [24]	39	2.6 × 1.6		IDC		MT	A	N	ER-PR-				R
										HER2-				
15	Khandelwal *et al*., 2009 [25]	35	2 × 3	2	IDC		BCS	A	P	ER+PR+	Yes	Yes	Yes	
										HER2-				
16	Iyengar *et al*., 2009 [26]	43	1.8	24	IDC		MT			ER-PR-				
										HER2+				
17	Rao *et al*., 2010 [27]	30	4 × 2	1	IDC		MT	A	P	ER-PR-	Yes	No	Yes	
										HER2-				
18	Petersson *et al*., 2010 [28]	49	3	48	IDC		Ex	S	N	ER+PR+				
										HER2-				
19	Kato *et al*., 2011 [29]	42	1.5		DCIS	TisN0M0	BCS	S	N					
20	Ooe *et al*., 2011 [30]	46	2.4 × 1.7	60	DCIS	TisN0M0	BCS	S	N	ER+PR+	No	Yes	Yes	
21	Tajima *et al*., 2011 [31]	60	1.9 × 1.6	3	ILC		BCS			ER+PR+				
										HER2-				
22	Abu-Rahmeh *et al*., 2012 [32]	69	5	180	IDC									Lung metastasis^b^
23	Jahan *et al*., 2012 [33]	55	5 × 4 × 3	240	IDC		MT							
24	Butler *et al*., 2012 [34]	46	0.8 × 0.8		ILC	T1bN0M0	Ex							
25	Present study	39	2.7 × 2.7	24	IDC	T1aN0M0	BCS	S	N	ER+PR+	No	Nr	Nr	
										HER2-				
26	Present study	31	3.5 × 3.4	84	IDC	T1aN1M0	MT	A	P	ER+PR+	Yes	Yes	No	
										HER2-				
27	Present study	30	1.5 × 1.4		DCIS	TisN0M0	BCS			ER+PR+	No	Yes	No	
										HER2-				
28	Present study	63	1.2	0.5	DCIS	TisN0M0	BCS	S	N	ER+PR+	No	Yes	Yes	
										HER2-				
29	Present study	48	0.9	3	DCIS	TisN0M0	Ex	S	N		No	No	No	
30	Present study	40	0.6	0	IDC	T1bN0M0	MT	S	N	ER +PR-	No	Yes	No	

^a^A, Axillary lymph node dissection level I/II/III; B, Benign; C/T, Chemotherapy; ER, Estrogen receptor; Ex, Excision; HER2, Human epidermal growth factor receptor 2 ; H/T, Hormone therapy; LN, Lymph node; M, Malignancy; MT, Mastectomy; N, Negative; Nr, Patient refuse; P, Positive; PR, Progesterone receptor; R, Recurrence; R/T, Radiotherapy; S, Sentinel lymph node dissection; TNM, Tumor, node, metastasis. ^b^Lung metastasis at diagnosis.

**Figure 2:**
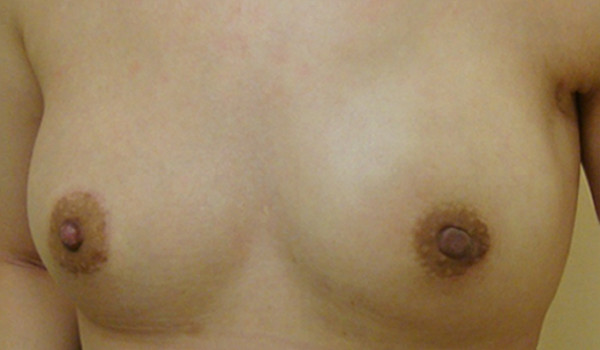
**Postoperative photograph of patient 26.** This patient had centrally located breast cancer arising from a fibroadenoma diagnosed before surgery. She was treated with endoscopically assisted nipple-sparing mastectomy combined with immediate breast reconstruction with a gel implant. Photograph was taken 3 months after surgery.

Twenty (66.7%) of thirty patients with BcaFad received breast-conserving surgery (BCS), and nine other patients (30.0%) received mastectomies. One (patient 26) of these nine mastectomy patients received immediate breast reconstruction (Figure 2). Furthermore, five (23.8%) of twenty-one patients with the diagnosis of lymph node status had lymph node metastasis. Most patients were diagnosed with IDC (16 cases, 53.3%), followed by DCIS (7 cases, 23.3%), LCIS (5 cases, 16.7%) and ILC (4 cases, 13.3%) (Table 2). Among these patients, only one patient (patient 2) had both LCIS and IDC and another (patient 5) had both DCIS and LCIS. The TNM stages recorded were major in TisN0M0, T1aN0M0 stage to T1bN0M0. A T1aN1M0 tumor was also noted.

**Table 2:** **Demographic and clinical features** ^**a**^

Characteristics ( ***N*** =30)	Data
Mean age (range), yr	46.9 (27 to 80)
Mean tumor size (range), cm	2.46 (0.8 to 5.1)
Female gender, *n* (%)	30 (100%)
Mean tumor follow-up duration (range) months	61.2 (0.5 to 600)
Histology^b^ (*N* =30), *n* (%)	
DCIS	7 (23.3%)
LCIS	5 (16.7%)
IDC	16 (53.3%)
ILC	4 (13.3%)
TNM stage (*N* =30), *n* (%)	
TisN0M0	10 (33.3%)
T1aN0M0	1 (3.3%)
T1bN0M0	3 (10.0%)
T1aN1M0	1 (3.3%)
Unclear	15 (30%)
Operation method (*N* =30), *n* (%)	
BCS	20 (66.7%)
Mastectomy	9 (30.0%)
No data	1 (3.3%)
Lymph node dissection (*N* =30), *n* (%)	
Axillary clearance	8 (26.7%)
Sentinel lymph node dissection	11 (36.7%)
Not done	1 (3.3%)
No data	10 (33.3%)
Lymph node (*N* =30), *n* (%)	
Metastasis	5 (16.7%)
Negative	16 (53.3%)
No data	9 (30.0%)
Receptor status	
ER + (*n* =16)	11 (68.8%)
PR + (*n* =16)	10 (62.5%)
HER2+ (*n* =10)	1 (10.0%)
Further management	
Chemotherapy (*n* =10)	4 (40.0%)
Hormone therapy (*n* =10)	6 (60.0%)
Radiation therapy (*n* =11)	5 (45.5%)
Outcome	
Average follow-up (range), months	21.9 (5 to 59)
Recurrence (*n* =16)	1 (6.3%)
Distant metastasis^c^ (*n* =16)	1 (6.3%)

^a^BCS, Breast-conserving surgery; DCIS, Ductal carcinoma *in situ*; ER, Estrogen receptor; HER2, Human epidermal growth factor receptor 2; IDC, Invasive ductal carcinoma; ILC, Invasive lobular carcinoma; LCIS, Lobular carcinoma *in situ*; PR, Progesterone receptor; TNM, Tumor, node, metastasis. ^b^One patient had both LCIS and IDC, and another had both DCIS and LCIS. ^c^Distant metastasis was noted at cancer diagnosis.

Regarding hormone receptor status and HER2 status, there were only 16 patients (53.3%) with data mentioned about ER and PR status. Eleven (68.8%) of these sixteen patients had ER + breast cancer, and ten patients (62.5%) had PR + cancer. Of ten patients with known HER2 status, only one patient (10.0%; patient 16) was found to have HER2-overexpressing breast cancer. Among 11 patients with hormone receptor–positive BcaFad, 6 patients (54.5%) received tamoxifen treatment. Four (80%) of five patients with lymph node metastasis received chemotherapy.

Nine patients were treated with mastectomy, and another twenty patients were treated with BCS. However, only four (20.0%) of the twenty patients who underwent BCS received radiotherapy [15, 19, 25]. Patient 17 received radiotherapy to the chest wall following a mastectomy performed for lymph node metastasis [27]. Of the four BCS patients with IDC, only one (25.0%) received radiation therapy. In addition, among six BCS patients with DCIS, only two (33.3%) received radiotherapy. Of fifteen patients with descriptions of outcome and distant metastasis, only one (patient 22) was found to have lung metastasis at diagnosis. Another patient (patient 14) had a local recurrence 5 months after surgery.

## Discussion

It is very unusual for a carcinoma of the breast to arise within a fibroadenoma. In this report, we present our 6 cases together with 24 previously reported cases for a collective case analysis. Among these 30 cases, the mean age at diagnosis of BcaFad was 46.9 years, which was older than that at diagnosis of benign fibroadenoma (age range from 20s to 30s) [5, 35]. The mean duration from finding a fibroadenoma to surgical excision was 61.2 months (range from 0.5 to 600 months). This long duration can be explained by the large size of the breast tumors (mean 2.46 cm) in this group of patients. This tumor size refers to the diameter of the fibroadenoma in which the malignant component was identified. In our present study, most BcaFads were predominantly found to be small cancers in relatively large fibroadenomas. However, the tumor size was not reported in most of the reviews we included, so we could not present the actual pathologic tumor size in each individual (Tables 1 and 2). Half of the data did not contain details regarding TNM stage; the other half were for stages TisN0M0, T1aN0M0, T1bN0M0 and T1aN1M0. However, we could assume that most of the limited data were for early breast cancer according to the descriptions of histology.

According to our collective case analysis, the major histological type of BcaFad was IDC (53.3%), followed by DCIS (23.3%), LCIS (16.7%) and ILC (13.3%) (Table 2). These findings are not consistent with previous reports that BcaFads were mainly carcinomas *in situ* (66.9% LCIS and 12.4% DCIS), followed by invasive carcinoma (11% IDC and 3.4% ILC) [9]. This discrepancy might be due to selection bias. Our present study is a collective analysis of case reports in the literature. Patients with invasive carcinoma inside fibroadenoma are rare and tend to be presented as case reports. In our six cases, the cancerous parts of the tumors were limited within the capsule of the underlying fibroadenoma without extension to peripheral breast tissue. In those BcaFad with DCIS, the DCIS was limited inside the fibroadenoma without invasion into the adjacent nonfibroadenoma breast tissue. In the LCIS-containing tumors, three patients had pure LCIS, one patient had both IDC and LCIS and two patients had both ILC and LCIS. None of them were found to have bilateral breast cancer. In our present analysis, we found that LCIS may be associated with invasive breast cancer.

In this study, 20 (66.7%) of 30 patients with BcaFad could be managed by BCS, and another 9 patients (30.0%) received mastectomies. The treatment principle for benign fibroadenomas is surgical excision with a thin ring of benign breast tissue [5], and breast conservation is usually feasible. BcaFad is usually found incidentally during the pathologic check when the breast tumor is being excised. This could explain why most of these patients (66.7%) received BCS as their surgical treatment. This breast-conserving rate is compatible with the reported data (19.6-58.0%) in the management of breast cancer with the same tumor size in the NASBP B-06 trial (National Surgical Adjuvant Breast and Bowel Project) and the National Cancer Database in the United States [36, 37].

Another reason for the high breast-conserving rate of the patients in this group is that most fibroadenomas are well-defined, with a "capsule" [2, 5]. If the initial resection margin is free of cancer or only LCIS is inside the fibroadenoma, then a tumorectomy or lumpectomy alone may be sufficient [15, 25, 38]. These facts could explain why the high rate of patients with BcaFad received BCS in this study. If the resection margin is involved or close, however, then a further wide excision may be needed [7].

Mastectomy (30.0% in this study) may still be needed in some circumstances—usually in large, multifocal or central located tumor [39]. One of our six patients (patient 26) received an endoscopically assisted, nipple-sparing mastectomy combined with immediate breast reconstruction using a gel implant because of the large size and central location of the breast tumor (Figure 2). Owing to the favorable prognosis, mastectomy with immediate breast reconstruction can be considered in patients with BcaFad when a mastectomy is needed.

Radiotherapy can effectively reduce local recurrence, and this reduction in disease relapse may translate to prolonged overall survival [11, 12, 40]. Nowadays, there is an established rule that, in order to decrease the risk of local recurrence, radiotherapy should be delivered to breast cancer patients receiving BCS [10, 13]. Should patients with BcaFad have to receive radiotherapy after partial mastectomy? Tiu *et al*. [41] stated that BcaFad behaves like breast cancer at the same stage and that therefore the treatment should follow the same modality. However, only four (20.0%) of the BCS patients received radiotherapy according to our collective case analysis [15, 19, 25]. Of four BCS patients with IDC, only one patient (25.0%; patient 15) received radiation therapy. In addition, among six BCS patients with DCIS, only two (33.3%) received radiotherapy.

According to our collective case analysis in this study, it seemed as though not all surgeons agreed that radiotherapy should be delivered to patients with BcaFad after BCS. To date, to our knowledge, no randomized controlled trials or large cohort studies have been conducted to answer this question. According to our literature review (Figure 1), fewer than 250 patients with BcaFad have been reported to date, and only 8 of them were reported to have received radiotherapy [14, 19, 25, 27, 30]. Whether radiotherapy is necessary for BcaFad patients after BCS is still unknown; more solid evidence is needed to establish the role of radiotherapy for this particular group of patients. To the best of our knowledge, radiotherapy seems optional rather than mandatory for BcaFad patients after partial mastectomy.

Owing to the limitations of a literature review, hormonal and HER2 receptor status was incompletely reported. In our collective case analysis, the incidence of hormone receptor–positive BcaFad was 68.8% ER + and 62.5% PR+, which is compatible with of the incidence in common types of breast cancer (range, approximately 60% to 90%) [42, 43]. However, the rate of HER2 overexpression was only 10.0%, which is a little lower than that of common types of breast cancer (approximately 15% to 25%) [44–46].

According to reports in the literature, most BcaFad cases are carcinoma *in situ* (79.3%), and less than 15% are invasive carcinomas [9]. BcaFad with lymph node metastasis is unusual, but can still occur [15, 19, 25, 27, 47, 48]. A sentinel lymph node biopsy should be performed if there is a pathologic confirmation of the presence of invasive carcinoma of BcaFad. Furthermore, axillary lymph node dissection should be performed in the presence of lymph node metastasis. Lymph node metastasis, rather than tumor size or hormone status, is the main concern underlying the use of chemotherapy for patients with BcaFad [19, 25, 27, 49].

The prognosis for patients with BcaFad was reported to be more favorable than that in other common types of breast cancer [19, 25]. In our collective case analysis, among 15 patients with descriptions of long-term results, 1 patient (6.67%, patient 14) had local recurrence 5 months after surgery [24] and another (patient 22) was found to have lung metastasis [32] when diagnosed with BcaFad. Death caused by BcaFad was uncommon, but still can occur in cases of invasive carcinomas at a late stage [50, 51].

## Conclusions

BcaFad is an infrequent malignancy and carries a favorable prognosis. Most BcaFads can be managed with BCS, and radiotherapy can be delivered optionally even when patient has undergone BCS. A sentinel lymph node biopsy should be performed if invasive carcinoma is present. In the presence of lymph node metastasis, chemotherapy should be considered to prevent distant metastasis.

## Authors’ information

YTW, STC, DRC, SJK and HWL: Comprehensive Breast Cancer Center, Changhua Christian Hospital, Changhua, Taiwan. YTW: Department of Surgery, Sinying Hospital, Ministry of Health and Welfare, Tainan, Taiwan. CJC: Department of Surgical Pathology, Changhua Christian Hospital, Changhua, Taiwan. YLK: Department of Surgery, National Cheng-Kung University Hospital, Tainan, Taiwan. LMT: Department of Surgery, Taipei Veterans General Hospital, Taipei, Taiwan.

## Abbreviations

BcaFad: Breast cancer arising within fibroadenoma
BCS: Breast-conserving surgery
DCIS: Ductal carcinoma *in situ*
ER: Estrogen receptor
IDC: Invasive ductal carcinoma
HER2: Human epidermal growth factor receptor 2
ILC: Invasive lobular carcinoma
LCIS: Lobular carcinoma *in situ*
PR: Progesterone receptor.
